# Associations of Health-Related Quality of Life, Fear of Falling and Objective Measures of Physical Function with Bone Health in Postmenopausal Women with Low Bone Mass

**DOI:** 10.3390/jcm8091370

**Published:** 2019-09-02

**Authors:** Anoohya Gandham, Lachlan B. McMillan, Carrie-Anne Ng, Ludovic Humbert, Maxine P. Bonham, Ayse Zengin, Peter R. Ebeling, David Scott

**Affiliations:** 1 Department of Medicine, School of Clinical Sciences at Monash Health, Monash University, Clayton, Victoria 3168, Australia; 2 Musculoskeletal Unit, Galgo Medical, 08036 Barcelona, Spain; 3 Department of Nutrition, Dietetics and Food, Monash University, Notting Hill, Victoria 3168, Australia; 4 Department of Medicine and Australian Institute of Musculoskeletal Science, Melbourne Medical School-Western Campus, The University of Melbourne, St Albans, Victoria 3010, Australia

**Keywords:** osteoporosis, postmenopausal women, physical function, bone, health-related quality of life

## Abstract

Health-related quality of life (HRQoL) and physical function deteriorate with age and may adversely impact bone health in older adults. We determined associations of objective measures of physical function and HRQoL with bone health in postmenopausal women with low areal bone mineral density (aBMD). Fifty postmenopausal women (64.4 ± 7.7 years old, mean ± standard deviation) with low spine, hip or femoral neck aBMD (T- or Z-score < −1.0) on dual-energy X-ray absorptiometry (DXA) participated. Femoral surface BMD, trabecular, integral and cortical volumetric BMD (vBMD) measurements were obtained using 3D-SHAPER software on DXA. Distal tibial vBMD and microarchitecture were assessed using high-resolution peripheral quantitative computed tomography (HRpQCT). Participants completed self-administered EuroQol-5D (EQ-5D) and modified falls efficacy scale (MFES) questionnaires, and physical function assessments. Stair climb power was positively associated with bone parameters at the hip, femoral neck, and distal tibia (all *p* < 0.05) in multivariable linear regression. EQ-5D demonstrated no significant associations with bone parameters and MFES was positively associated only with distal tibial cortical vBMD and cortical von Mises stress (both *p* < 0.05). Objective measures of physical function, particularly muscle power, are more consistently associated with bone parameters compared with self-administered HRQoL questionnaires.

## 1. Introduction

Osteoporosis is an age-associated disease largely affecting postmenopausal women due to a steep decline in estrogen levels following menopause [1,2]. Osteoporosis is defined as low bone mineral density (BMD) and bone microarchitecture increasing bone fragility and the risk of fracture [3,4]. The current clinical gold standard for diagnosing osteoporosis is a dual-energy X-ray absorptiometry (DXA) scan which measures areal BMD (aBMD) [1,5]. Although DXA has many advantages, there are a number of limitations, such as space restrictions in a clinical setting and equipment expenses. Hence, the development of cost-effective but reliable methods for identifying individuals at increased risk for poor bone health is important in reducing the burden of osteoporosis.

Age-related declines in physical function, particularly in postmenopausal women, are sequentially associated with loss of bone and bone mass [6]. Declining physical activity levels with age contribute to bone fragility and an increasing fracture risk in older adults [6,7]. Individuals with better physical function are more capable of engaging in physical activity, which has a protective effect on bone health through mechanical loading [8]. Physical performance assessments can easily be incorporated into a clinical environment given they are generally feasible and have been recommended for risk assessment and diagnosis for a range of conditions, including sarcopenia [8,9]. It is possible that they may also have utility for prediction of bone loss in older adults and may be useful for clinicians to identify individuals at risk of poor bone health [9,10]. 

Health-related quality of life (HRQoL) comprises aspects of physical, emotional and mental-wellbeing which are known to decrease with age [11]. HRQoL instruments are useful and inexpensive assessment tools for overall health status [11,12]. While osteoporosis negatively affects HRQoL in older adults, it is also possible that declines in HRQoL contribute to reductions in bone health, in a bidirectional manner. Fear of falling may also be associated with HRQoL and increases with age resulting in a lack of confidence in performing daily tasks, particularly in older women [12]. Both poor HRQoL and fear of falling may, therefore, be associated with bone health in older adults and could be clinically relevant self-reported measures to identify individuals at increased risk of osteoporosis [13]. 

The aim of the present study was to determine the associations between self-reported HRQoL, fear of falling, and objective measures of physical function with BMD and bone microarchitecture in postmenopausal women with low aBMD.

## 2. Methods

### 2.1. Study Design and Participants

This analysis utilized baseline data from an exercise intervention conducted at the Monash Health Translation Precinct in Melbourne, Australia. Fifty community-dwelling postmenopausal women residing in Melbourne, Australia were recruited for this study. Recruitment of participants was achieved through advertisements in the local community, hospitals, businesses, and clubs. Inclusion criteria included: T- or Z-score at spine, hip or femoral neck <−1.0, body mass index (BMI) <30 kg/m^2^, <150 min of self-reported moderate to vigorous physical activity per week, and English-speaking. The study (ANZCTR number: ACTRN12618000101280) was approved by Monash Health Human Research Ethics Committee (HREC/16/MonH/364) and all participants provided written informed consent prior to completing any study assessments. 

### 2.2. Questionnaires

At baseline, participants completed self-administered questionnaires including questions on demographics, medication use, disease history, smoking status, and physical activity. HRQoL was measured using the EuroQol-5D (EQ-5D) [12]. The EQ-5D consists of five HRQoL dimensions: Mobility, self-care, usual activities, pain/discomfort, and anxiety/depression. Participants selected one of three responses for each of the dimensions based on which an EQ-5D score was derived to measure the HRQoL status. Scores of the EQ-5D questionnaire range from 1 to 3, where 1 indicates no problems, 2 indicates some problems, and 3 indicates extreme problems/unable to perform that activity from which a total EQ-5D score (range: 5–15, higher scores indicate poorer HRQoL) has been derived. The modified falls efficacy scale (MFES) was used to assess fear of falls while completing certain tasks [14,15]. MFES consists of 14 questions related to a range of daily activities to determine confidence undertaking certain tasks (for example, get in/out of chair) [14]. Participants rated their level of confidence for each of the activities on a scale from 0 to 10, with 0 meaning not confident/not sure and 10 being completely confident/completely sure. The possible range of total scores in the MFES questionnaire was 0 to 140 (with higher scores indicating better confidence) [16].

### 2.3. Physical Function

Objective assessments of physical function were performed, including the stair climb power test (SCPT) and the short physical performance battery (SPPB). The SCPT was performed to evaluate leg muscle power and mobility performance [17]. Participants began the test from the base of a 10-stair flight of stairs and were asked to climb the stairs as fast as possible, in a safe manner. The time taken by the participants to ascend the flight of stairs was recorded in seconds using a stopwatch. We then calculated stair climb power using the following formula: power = force X velocity [17]. Force was calculated as the product of participant body mass (kg) and acceleration due to gravity (9.8 m/s^2^). Velocity was calculated from the vertical height of stairs (1.75 m) divided by stair climb time (s). The SPPB test consists of gait speed, chair stand, and standing balance assessments and provides a composite SPPB score [18,19]. For the chair stand test, participants were required to stand up straight from a seated position five times as quickly as possible and the time taken was recorded. To complete the gait speed test, participants were required to walk 2.44 m at a comfortable and normal pace. Gait speed was calculated by distance (walking course 2.44 m) and time taken to complete the test (s). For the standing balance tests, the participants were required to hold two positions: Semi-tandem and full-tandem, for 10 s. The semi-tandem stand involves placing the preferred foot in front of the other with the side of the heel from the front foot touching the first toe of the other foot. The full-tandem stand is performed by placing the preferred foot in front of the other and the back of the heel on the front foot touching the toes of the back foot. A total score out of 12 (higher scores indicate better physical function) was calculated based on the performance of the three tests: Chair stand, gait speed, and standing balance assessments [18]. 

### 2.4. Anthropometric, Bone and Blood Biochemistry Parameters

Participants attended the clinic appointment following an overnight fast of ≥ 10 h. A 20 mL blood sample was collected by a qualified phlebotomist, and serum samples were analyzed for 25-hydroxyvitamin D (25(OH)D) using the DiaSorin Liaison (DiaSorin Inc, Stillwater, MN, USA) 25(OH)D assay, a direct competitive chemiluminescent immunoassay. 

Height (m) and weight (kg) were measured with footwear, headwear, and heavy items of clothing removed. Height was measured using a portable stadiometer (Seca 213 wall-mounted stadiometer, Seca, Hamburg, Germany), weight was measured using an electronic scale (Seca 804, Germany) and body mass index (BMI; kg/m^2^) was calculated.

Each participant had a DXA scan (Hologic Discovery A, Hologic, Bedford, MA, USA), where aBMD and bone mineral content (BMC) were assessed at the total hip, femoral neck, and lumbar spine using Hologic Apex software version 5.6.0.2. Osteoporosis was classified as T-score ≤ −2.5 and osteopenia as T-score between −1 and −2.5, in line with guidelines from World Health Organization [20]. Lumbar spine trabecular bone score (TBS) was assessed using TBS iNsight software version 3.0.2 (Medimaps, Geneva, Switzerland). Using an algorithm, the 3D-SHAPER software (Galgo Medical, Barcelona, Spain) was applied to two-dimensional DXA scans of the proximal femur to model the femoral shape and BMD distribution in three dimensions, and then derived estimates for trabecular, cortical, and integral volumetric BMD (vBMD) and surface BMD, a measure of fracture resistance [21]. 

High-resolution peripheral quantitative computed tomography (HRpQCT) scans (XtremeCT II, ScanCo, Switzerland) were performed to determine the integral bone quality and strength at the distal tibia [22,23]. Briefly, the non-dominant leg was placed in a cast and stabilized to prevent movement and subsequent artifacts. A scout scan was used to place the reference line at the tibial endplate. A subsequent scan of one hundred and sixty-eight parallel computed tomography slices, encompassing a 9.02 mm region of the distal tibia was performed with an isotropic resolution of <55 μm. HRpQCT scans were then analyzed using the manufacturer’s standard protocol, using software version 6.1. Trabecular and cortical bone parameters derived from HRpQCT included: Trabecular cross-sectional area (mm^2^), trabecular thickness (mm) trabecular separation (mm, the mean space between the trabeculae), cortical cross-sectional area (mm), cortical vBMD, cortical porosity (%, the segmented pore volume) and cortical periosteal perimeter (mm). [22,23]. Micro-finite element analysis was also performed to assess the effects of a simulated mechanical load to estimate trabecular and cortical von Mises stress (MPa), which is used for fracture prediction with lower values indicating greater fracture resistance, as well as estimated bone failure load (N) and bone stiffness (N/mm^2^) [24,25].

### 2.5. Accelerometer-Determined Physical Activity

Each participant was provided with an Actigraph GT3X-BT accelerometer (Actigraph, Pensacola, CA, USA) and instructed to wear the accelerometer at the side of the hip for seven consecutive days except during showering, water activities, and sleeping. A diary was also provided, to record the time of the day that the device was worn and removed, time spent outdoors, physical activity performed and any issues that affected their physical activity estimates (for example, health issues resulting in low physical activity). The accelerometer data was recorded at a rate of 50 Hz and transferred into ActiLife v6.13.3 software for analysis. Accelerometer activity was transformed into counts per minute (CPMs) with an activity threshold of 100 CPMs. The ActiLife software analyzed data in epoch lengths of 60 seconds. Physical activity was categorized based on the following Freedson adult cut points of 1 to 99 CPMs (sedentary), 100 to 1951 CPMs (light) and >1952 CPMs (moderate to vigorous physical activity, MVPA) [26]. MVPA was combined into a single variable due to the low amount of vigorous physical activity recorded in these postmenopausal women [27].

### 2.6. Statistical Analysis

All statistical analyses were performed using SPSS software version 25 (IBM, Chicago, IL, USA). Data related to HRQoL and physical function were non-normally distributed, and so Spearman’s correlations were performed to determine associations for self-reported HRQoL (EQ-5D and MFES) and objective measures of physical function (SCPT, SPPB and its components, with the exception of standing balance) with bone parameters. Multivariable linear regression analysis was performed to further investigate these associations after adjusting for confounders including age, MVPA, BMI, and 25(OH)D. For all analyses, *p*-values < 0.05 were considered statistically significant. 

## 3. Results

In total, 50 postmenopausal women were recruited with an age range of 49–82 years (Table 1). Mean BMI was in the normal range with no current smokers in this population and the most common self-reported comorbidities were hypertension and hypercholesterolemia. The majority (88%) of participants had sufficient 25(OH)D levels (≥50 nmol/L). Twenty-two (44%) participants had osteoporosis and twenty-seven (54%) had osteopenia, based on DXA T-scores. One 49-year-old participant had total hip and lumbar spine Z-scores of −1.5 and −0.9, respectively. No participants indicated extreme problems or an inability to perform activity in any of the five domains of the EQ-5D questionnaire. Most participants (62%) had the maximum MFES score of 140, indicating general good health based on the self-reported HRQoL and fear or falling questionnaires. Participants spent almost three-quarters of their accelerometer wear time in sedentary behavior, and only approximately 5% of their time performing moderate to vigorous intensity activity. Twenty-nine (56%) participants had an SPPB score of 12/12 and participants had a range of 176–385 W for the SCPT. 

**Figure 1:**
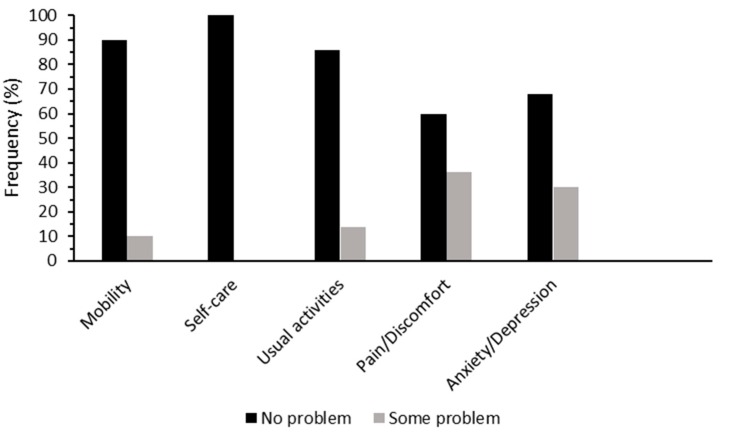
**Percentage of** postmenopausal women who had no problem (black) and some problems (grey) for the five dimensions of EuroQol-5D (EQ-5D).

Figure 1 presents the number of participants with either no problem or some problems for each of the five dimensions of EQ-5D, none reported extreme problems. For the self-care domain, all participants reported no problems. The pain/discomfort domain had the highest proportion of participants selecting some problems (36%).

Table 2 reports Spearman’s correlation coefficients for the self-reported EQ-5D and its domains, and MFES, with bone parameters. The EQ-5D domain of self-care was not included in this analysis as all participants selected the same response (score of 1). EQ-5D total score was not correlated with any of the bone parameters but the EQ-5D mobility domain was significantly negatively correlated with cortical vBMD at the tibia (*p* = 0.027). The pain/discomfort domain was significantly negatively correlated with trabecular vBMD (*p* = 0.019) and integral vBMD at the total hip (*p* = 0.045). MFES was significantly negatively correlated with hip BMC only (*p* = 0.024).

Spearman’s correlations are presented in Table 3 for relationships between objective measures of physical function and bone parameters. SCPT was significantly positively correlated with a majority of the DXA bone parameters including hip BMC and T-score, spine BMC and aBMD, lumbar spine TBS and femoral neck BMC, aBMD, and T-score, and was also significantly correlated with HRpQCT bone parameters, including trabecular area and cortical periosteal perimeter at the distal tibia. However, SCPT was significantly negatively associated with trabecular thickness, estimated bone failure load, and bone stiffness at the distal tibia. Total SPPB score was significantly positively correlated with both lumbar spine TBS and cortical von Mises stress at the distal tibia. Gait speed was significantly negatively correlated with trabecular thickness at the distal tibia while chair stand time was significantly negatively correlated with cortical area and cortical von Mises stress at the distal tibia. 

Table 4 presents coefficients from the multivariable linear regression model examining the associations of self-reported HRQoL and objective measures of physical function with bone parameters. After adjustment for confounders, EQ-5D had no significant associations with any of the bone parameters. However, MFES was significantly positively associated with cortical vBMD and cortical von Mises stress at the distal tibia. SCPT remained significantly positively associated with hip BMC, femoral neck BMC, aBMD and T-score, and trabecular area at the distal tibia and was also significantly negatively associated with trabecular thickness at the distal tibia. SPPB was significantly positively associated with cortical von Mises stress at the distal tibia. No associations were found between chair stand time and bone parameters however, gait speed was significantly negatively associated with trabecular thickness and trabecular von Mises stress at the distal tibia. 

## 4. Discussion

Our cross-sectional study in community-dwelling postmenopausal women with low aBMD demonstrates that an objective measure of muscle power is more consistently associated with bone parameters than other clinically relevant objective measures of physical performance, self-reported HRQoL, and fear of falling. This is the first study to compare associations of self-administered questionnaires and objectively measured physical function with bone health using advanced bone imaging. Our data suggest objective assessments, particularly the SCPT, may be used in screening individuals at risk of poor bone health in clinical settings. Given that physical assessments can be easily incorporated in clinical settings, they may serve as useful screening tools to help address current underdiagnosis of osteoporosis [28].

Total EQ-5D scores had no associations with any bone parameters. In contrast, a prior study of 325 women with mean age of 60 years demonstrated that osteoporotic women have poorer HRQoL as estimated by EQ-5D questionnaires, compared with healthy individuals of a similar age [29]. Additionally, a study including 222 participants with mean age of 79 years reported that those with osteoporosis had poorer HRQoL even compared with those with osteopenia, although the HRQoL in this study was measured using the quality of life questionnaire of the International Osteoporosis Foundation (QUALEFFO-41), which specifically measures osteoporosis-related HRQoL [30]. While we did not compare the women with osteopenia or osteoporosis in this study to a control group with normal aBMD, the lack of association between HRQoL and bone parameters in this population is potentially explained by the overall good general health in the current study where the majority of women had either no or few problems for each of the EQ-5D domains. Nonetheless, longitudinal studies with a larger sample size are required to determine whether poorer HRQoL is predictive of an accelerated decline in bone health in older age. It is possible that an osteoporosis-specific HRQoL instrument may be more sensitive in detecting such a relationship.

Despite the lack of association for total EQ-5D scores, the mobility and pain/discomfort domains of EQ-5D were associated with the bone parameters. A study of 1000 Korean women aged ≥60 years reported that individuals with osteoporosis had significantly higher scores in the mobility, pain/discomfort, and usual activities domains of EQ-5D [31]. The lack of association of usual activities HRQoL with bone health in the present study may relate to the fact that only a few women reported some difficulty with usual activities. It is possible that identification of women with reduced mobility and increased pain/discomfort could be a useful strategy to determine an increased osteoporosis risk.

Our study is the first we are aware of to demonstrate an association between higher MFES scores (indicating lower fear of falling) and better cortical bone health, including distal tibial cortical vBMD and cortical von Mises stress. These associations were significant even after adjusting for confounders, including physical activity, in multivariable linear regression analyses suggesting that other factors associated with fear of falling, perhaps including mobility and pain/discomfort, may influence bone health in women with low aBMD. A study of 140 participants reported that fear of falling is increased in older adults with osteoporosis [32]. However, in the current study, the majority of women with low aBMD had the maximum overall score of 140, indicating the lowest fear of falling. As demonstrated by the objective measures of physical function, and the fact that these women self-selected to participate in an exercise intervention, they likely represent a sample of the postmenopausal population with a relatively low falls risk. Furthermore, although knowledge of osteoporosis and awareness of a diagnosis increases fear of falls in older women [32], the majority of women in the current study were osteopenic rather than osteoporotic, and therefore, less susceptible to fear of falling. Regardless, the association of fear of falling with cortical bone parameters warrants further investigation.

Physical function is potentially an important predictor of fracture risk, largely due to its relationship with falls [33]. Previous studies have also suggested that improved physical performance is associated with better bone health. A study of 3041 older adults reported that physical performance was moderately associated with hip BMD and we previously reported in a study on over 3300 Swedish older adults that better performance in the timed up-and-go test was associated with better distal tibial trabecular vBMD assessed by standard peripheral quantitative computed tomography [34,35]. Surprisingly, chair stand time was not associated with any of the bone parameters in this study, and gait speed was significantly negatively associated with trabecular thickness and trabecular von Mises stress at the distal tibia. A study of 116 postmenopausal women observed that gait speed was positively associated with hip aBMD, but similarly found no associations between chair rise time and aBMD [36]. It is possible that gait speed has differing associations with bone compartments at different anatomical sites, given that the hip primarily comprises cortical rather than trabecular bone. A study of 1061 Japanese postmenopausal women reported that gait speed can be used in combination with lower extremity strength measurements as screening tools for low aBMD in postmenopausal women, but based on the findings of the present study, further research is required to explore its associations with bone microarchitectural parameters and vBMD [9]. Previous research has also demonstrated that physical performance tests can improve the predictive ability of existing fracture prediction tools, but the present study is the first we are aware of to indicate that a simple assessment of muscle power, rather than physical performance per se, is more consistently associated with bone health. Thus, future prospective studies should explore whether the addition of muscle power assessment improves fracture prediction compared with other functional assessments [37].

Stimulation of bone formation through resistance exercise is necessary for the maintenance of bone health due to the insufficient mechanical loading obtained from daily activities in older adults [38]. Furthermore, high-intensity resistance training has been shown to improve aBMD and enhance physical function in older women, and this exercise targeting muscle power may be superior to traditional resistance training for maximizing bone health in older age [39]. We observed that SCPT was positively associated with the distal tibial trabecular area, as well as with femoral neck and total hip aBMD. Our findings, therefore, support the concept that improving muscle power may be beneficial for maintaining bone health in older adults, although, as was observed for gait speed, SCPT was negatively associated with trabecular thickness at the distal tibia. It has been reported that increased mechanical loading through physical activity leads to changes to morphological parameters other than trabecular thickness [40], and trabecular number appears more important than trabecular thickness for maintaining bone strength [41,42]. Hence, a negative association may not be a clinically significant finding and it should also be noted that trabecular thickness is a derived measurement from HRpQCT rather than a directly measured bone parameter [23]. As a result, assessments of muscle power in clinical practice appears potentially useful for screening for poor bone health in postmenopausal women. 

The results of this study are subject to limitations including its cross-sectional design, which limits conclusions on causality of any observed associations. It is likely that poorer bone health contributes to functional decline, poor HRQoL, and fear of falling in postmenopausal women, but we did not ascertain whether previous falls and fractures influenced these relationships. The study had a relatively small sample size and it is unclear whether these results can be generalized to either men or populations with normal aBMD. Hence, longitudinal studies should be conducted in larger populations to confirm if objective measures of physical function are stronger and better predictors of decline in bone health compared with self-reported HRQoL and fear of falls questionnaires. The strengths of the study include a well-defined sample of women with poor bone health, the use of both objective and self-reported measures of physical function, and advanced imaging of bone including HRpQCT.

In conclusion, a clinical assessment of muscle power was more consistently associated with microarchitectural bone parameters compared with other measures of physical function, and self-administered HRQoL and fear of falling questionnaires, in postmenopausal women with low aBMD. In the absence of access to bone imaging devices such as DXA and HRpQCT, and given their concurrent association with risk of falls, it may be beneficial for clinicians to incorporate measures of muscle power in order to screen for poor bone health in older adults. Furthermore, interventions targeting improvements in muscle power may be effective in improving bone health in postmenopausal women at risk of osteoporosis.

## Figures and Tables

**Table 1:** Participant descriptive characteristics (*n* = 50).

	Mean ± Standard Deviation or Percentage
Age (years)	64.4 ± 7.7
Weight (kg)	61.5 ± 8.5
Height (cm)	161.3 ± 6.5
BMI (kg/m^2^)	23.6 ± 3.0
25(OH)D (nmol/L)	74.6 ± 25.1
aBMD	
Total hip (g/cm^2^) Femoral neck (g/cm^2^) Lumbar spine (g/cm^2^)	0.765 ± 0.081 0.636 ± 0.070 0.809 ± 0.112
BMC	
Total hip (g) Femoral neck (g) Lumbar spine (g)	26.582 ± 3.708 3.207 ± 0.388 43.585 ± 10.916
Smoking status	
Current (%) Former (%) Never (%)	0 22 78
HRQoL and fear of falling	
EQ-5D score (out of 15) MFES score (out of 140)	5 (5.0, 6.3) # 140 (134.8, 140) #
Physical activity	
Sedentary time physical activity (%) Light-intensity physical activity (%) Moderate-intensity physical activity (%) Vigorous-intensity physical activity (%) Very vigorous-intensity physical activity (%)	73.3 ± 6.0 21.7 ± 4.7 4.8 ± 2.4 0.2 ± 0.4 0.004 ± 0.01
Objective measures of physical function	
SPPB (out of 12) SCPT (W)	12 (11, 12) # 277.2 (229.1, 335.5)
Self-reported comorbidities	
Hypertension (%) Hypercholesterolemia (%) Thrombosis (%) T2DM (%) Asthma (%) Bronchitis/emphysema (%) Any cancer (%)	22 28 2 4 14 2 6

All data are expressed as mean ± SD or frequency (%) unless otherwise specified. # median (IQR). Abbreviations: BMI, body mass index; 25-OHD, 25-hydroxyvitamin D; aBMD, areal bone mineral density; BMC, bone mineral content; HRQoL, Health-related quality of life; EQ-5D, EuroQol-5D; MFES, modified falls efficacy scale; IQR, interquartile range; SPPB, short physical performance battery; SCPT, stair climb power test; T2DM, type 2 diabetes mellitus.

**Table 2:** Spearman’s correlations between self-reported measurements and bone parameters.

	EQ-5D	Mobility	Usual	Pain	Anxiety	MFES
DXA bone parameters						
Hip BMC (g)	0.264	0.132	0.222	0.158	0.269	−0.318 *
Hip aBMD (g/cm^2^)	0.002	−0.021	0.066	−0.109	0.031	−0.196
Hip T-score	−0.008	−0.036	0.060	−0.103	0.036	−0.173
Spine BMC (g)	−0.056	−0.081	−0.034	−0.096	−0.044	0.041
Spine aBMD (g/cm^2^)	−0.187	−0.076	−0.074	−0.165	−0.157	0.007
Spine T-score	−0.149	−0.057	−0.062	−0.155	−0.128	−0.015
LS TBS	−0.033	−0.039	−0.056	−0.045	0.103	0.062
FN BMC (g)	0.064	0.049	0.026	0.000	0.056	0.015
FN aBMD (g/cm^2^)	−0.066	−0.025	−0.078	−0.118	−0.053	0.095
FN T-score	−0.050	−0.017	−0.062	−0.126	−0.023	0.070
DXA 3D bone parameters						
Surface BMD (mg/cm^3^)	0.084	−0.043	0.037	−0.045	0.036	−0.157
Trabecular vBMD (mg/cm^3^)	−0.259	−0.076	−0.049	−0.342 *	−0.149	−0.022
Integral vBMD (mg/cm^3^)	−0.194	−0.086	−0.016	−0.294 *	−0.149	−0.078
Cortical vBMD (mg/cm^3^)	−0.049	−0.095	0.058	−0.142	−0.069	−0.198
Mean cortical thickness (mm)	0.149	−0.010	0.033	0.013	0.119	−0.087
HR-pQCT bone parameters at distal tibia						
Trabecular area (mm^2^)	0.080	0.173	0.054	0.099	−0.072	−0.163
Trabecular thickness (mm)	0.061	0.113	0.112	−0.081	0.045	0.014
Trabecular separation (mm)	0.019	0.035	−0.086	−0.103	0.080	−0.048
Trabecular VMS (MPa)	−0.187	−0.043	−0.091	−0.245	−0.031	0.111
Cortical Area (mm^2^)	−0.023	−0.146	−0.182	−0.023	−0.011	0.081
Cortical vBMD (mg/cm^3^)	−0.139	−0.312 *	−0.104	−0.073	−0.011	0.133
Cortical periosteal perimeter (mm)	0.022	0.148	0.110	0.042	−0.180	−0.142
Cortical porosity (%)	−0.013	0.092	−0.092	−0.020	−0.005	0.044
Cortical VMS (MPa)	−0.263	−0.248	−0.198	−0.261	−0.041	0.187
Estimated bone failure load (N)	0.081	0.157	0.095	0.099	−0.024	−0.169
Bone stiffness (N/mm)	−0.092	−0.143	−0.091	−0.109	0.005	0.155

All data are presented as Spearman’s correlation coefficient (*p*-value). Abbreviations: DXA, dual-energy X-ray absorptiometry; HRpQCT, high-resolution peripheral quantitative computed tomography; EQ5D, EuroQol-5D; MFES, modified falls efficacy scale; SPPB, short physical performance battery; BMC, bone mineral content; aBMD, areal bone mineral density; LS, lumbar spine; TBS, trabecular bone score; FN, femoral neck; VMS, von Mises stress. * Correlation is significant at the 0.05 level (2-tailed).

**Table 3:** Spearman’s correlations between objective measurements and bone parameters.

	Gait Speed (m/s)	Stair Climb Power (W)	Chair Stand Time (s)	SPPB
DXA bone parameters				
Hip BMC (g) Hip aBMD (g/cm^2^) Hip T-score Spine BMC (g) Spine aBMD (g/cm^2^) Spine T-score LS TBS FN BMC (g) FN aBMD (g/cm^2^) FN T-score	0.272 0.234 0.226 0.236 0.175 0.152 0.159 0.220 0.181 0.168	0.390 ** 0.249 0.296 * 0.395 ** 0.317 * 0.271 0.344 * 0.405 ** 0.328 * 0.347 *	−0.043 −0.172 −0.163 −0.191 −0.107 −0.116 −0.262 −0.007 −0.082 −0.089	0.106 0.211 0.190 0.026 −0.083 −0.062 0.299 * 0.092 0.002 0.032
DXA 3D bone parameters				
Surface BMD (mg/cm^3^) Trabecular vBMD (mg/cm^3^) Integral vBMD (mg/cm^3^) Cortical vBMD (mg/cm^3^) Mean cortical thickness (mm)	0.204 0.188 0.200 0.199 0.203	0.197 0.111 0.118 0.068 0.279	−0.064 −0.271 −0.258 −0.136 −0.029	0.124 0.245 0.221 0.132 0.139
HR-pQCT bone parameters at distal tibia				
Trabecular area (mm^2^) Trabecular thickness (mm) Trabecular separation (mm) Trabecular VMS (Mpa) Cortical Area (mm^2^) Cortical vBMD (mg/cm^3^) Cortical periosteal perimeter (mm) Cortical porosity (%) Cortical VMS (MPa) Estimated bone failure load (N) Bone stiffness (N/mm)	0.151 −0.413 ** −0.196 −0.213 0.063 0.071 0.091 −0.018 0.118 −0.066 0.042	0.342 * −0.314 * −0.220 0.024 0.090 0.146 0.329 * −0.208 −0.036 −0.314 * −0.314 *	0.153 0.097 0.058 −0.192 −0.341 * −0.186 0.116 −0.093 −0.298 * 0.217 −0.217	−0.045 −0.109 0.119 0.018 0.087 0.229 −0.069 −0.151 0.331 * −0.106 0.079

All data are presented as Spearman’s correlation coefficient (p-value). Abbreviations: DXA, dual-energy X-ray absorptiometry; HRpQCT, high-resolution peripheral quantitative computed tomography; SPPB, short physical performance battery; BMC, bone mineral content; aBMD, areal bone mineral density; LS, lumbar spine; TBS, trabecular bone score; FN, femoral neck; VMS, von Mises stress. ** Correlation is significant at the 0.01 level (2-tailed). * Correlation is significant at the 0.05 level (2-tailed).

**Table 4:** Multivariable linear regression analysis examining associations between self-reported, objective measurements, and bone parameters.

	EQ-5D Score	MFES Score	Gait Speed (m/s)	Stair Climb Power (W)	Chair Stand Time (s)	SPPB
DXA bone parameters						
Hip BMC (g)	0.218 (−0.072, 0.509)	−0.175 (−0.467, 0.117)	0.266 (−0.030, 0.562)	**0.485 (0.175, 0.795)**	−0.157 (−0.467, 0.153)	0.016 (−0.344, 0.376)
Hip aBMD (g/cm^2^)	−0.012 (−0.327, 0.303)	0.032 (−0.282, 0.346)	0.265 (−0.050, 0.580)	0.263 (−0.093, 0.618)	−0.271 (−0.592, 0.051)	0.243 (−0.130, 0.616)
Hip T-score	−0.026 (−0.339, 0.288)	0.038 (−0.274, 0.351)	0.254 (−0.061, 0.569)	0.239 (−0.112, 0.591)	−0.251 (−0.568, 0.066)	0.212 (−0.161, 0.586)
Spine BMC (g)	−0.106 (−0.410, 0.198)	0.119 (−0.184, 0.421)	0.219 (−0.089, 0.527)	0.288 (−0.055, 0.630)	−0.162 (−0.480, 0.156)	−0.107 (−0.475, 0.261)
Spine aBMD (g/cm^2^)	−0.263 (−0.566, 0.040)	0.204 (−0.102, 0.510)	0.145 (−0.177, 0.466)	0.220 (−0.137, 0.576)	−0.136 (−0.464, 0.191)	−0.100 (−0.478, 0.277)
Spine T-score	−0.252 (−0.562, 0.058)	0.211 (−0.101, 0.523)	0.134 (−0.195, 0.463)	0.224 (−0.135, 0.583)	−0.165 (−0.494, 0.164)	−0.063 (−0.450, 0.324)
LS TBS	−0.020 (−0.290, 0.249)	0.098 (−0.169, 0.364)	−0.047 (−0.326, 0.231)	0.034 (−0.278, 0.346)	−0.046 (−0.329, 0.238)	0.052 (−0.274, 0.377)
FN BMC (g)	−0.018 (−0.322, 0.286)	0.175 (−0.123, 0.473)	0.294 (−0.006, 0.594)	**0.458 (0.136, 0.780)**	−0.165 (−0.481, 0.151)	0.268 (−0.090, 0.625)
FN aBMD (g/cm^2^)	−0.126 (−0.434, 0.182)	0.250 (−0.050, 0.550)	0.198 (−0.117, 0.513)	**0.353 (0.010, 0.696)**	−0.205 (−0.526, 0.116)	0.292 (−0.071, 0.656)
FN T-score	−0.121 (−0.431, 0.190)	0.245 (−0.057, 0.547)	0.188 (−0.131, 0.506)	**0.351 (0.011, 0.692)**	−0.206 (−0.525, 0.113)	0.306 (−0.059, 0.671)
DXA 3D bone parameters						
Surface BMD (mg/cm^3^)	−0.007 (−0.328, 0.313)	0.001 (−0.308, 0.309)	0.211 (−0.102, 0.524)	0.191 (−0.163, 0.544)	−0.122 (−0.445, 0.202)	0.121 (−0.251, 0.492)
Trabecular vBMD (mg/cm^3^)	−0.246 (−0.561, 0.070)	0.148 (−0.161, 0.457)	0.145 (−0.176, 0.466)	0.075 (−0.287, 0.437)	−0.252 (−0.573, 0.068)	0.315 (−0.051, 0.680)
Integral vBMD (mg/cm^3^)	−0.200 (−0.520, 0.121)	0.095 (−0.218, 0.408)	0.151 (−0.171, 0.474)	0.054 (−0.311, 0.419)	−0.235 (−0.559, 0.089)	0.238 (−0.136, 0.611)
Cortical vBMD (mg/cm^3^)	−0.086 (−0.416, 0.243)	−0.053 (−0.375, 0.268)	0.210 (−0.118, 0.538)	0.068 (−0.301, 0.437)	−0.170 (−0.502, 0.162)	0.072 (−0.318, 0.463)
Mean cortical thickness (mm)	0.077 (−0.236, 0.389)	0.041 (−0.264, 0.347)	0.191 (−0.121, 0.502)	0.243 (−0.100, 0.585)	−0.069 (−0.388, 0.249)	0.153 (−0.215, 0.521)
HR-pQCT bone parameters at distal tibia						
Trabecular area (mm^2^)	0.161 (−0.150, 0.472)	−0.215 (−0.521, 0.092)	0.180 (−0.141, 0.501)	**0.444 (0.106, 0.781)**	0.155 (−0.173, 0.484)	−0.044 (−0.425, 0.336)
Trabecular thickness (mm)	0.076 (−0.249, 0.402)	−0.052 (−0.376, 0.273)	**−0.439 (−0.745, −0.132)**	**−0.387 (−0.745, −0.029)**	0.089 (−0.254, 0.431)	0.002 (−0.393, 0.396)
Trabecular separation (mm)	0.311 (−0.002, 0.624)	−0.052 (−0.376, 0.273)	−0.115 (−0.452, 0.222)	−0.286 (−0.654, 0.082)	0.002 (−0.342, 0.347)	0.336 (−0.044, 0.717)
Trabecular VMS (MPa)	−0.221 (−0.539, 0.097)	0.078 (−0.245, 0.401)	**−0.322 (−0.643, −0.002)**	−0.200 (−0.567, 0.166)	−0.175 (−0.509, 0.158)	0.028 (−0.366, 0.421)
Cortical Area (mm^2^)	−0.081 (−0.400, 0.241)	0.218 (−0.097, 0.534)	0.043 (−0.290, 0.375)	0.033 (−0.340, 0.405)	−0.279 (−0.606, 0.049)	−0.071 (−0.459, 0.317)
Cortical vBMD (mg/cm^3^)	−0.219 (−0.533, 0.094)	**0.339 (0.037, 0.641**	0.051 (−0.281, 0.382)	0.062 (−0.308, 0.433)	−0.168 (−0.501, 0.166)	0.111 (−0.274, 0.497)
Cortical periosteal perimeter (mm)	0.112 (−0.196, 0.419)	−0.212 (−0.514, 0.090)	0.085 (−0.234, 0.405)	0.447 (0.116, 0.777)	0.081 (−0.244, 0.407)	−0.114 (−0.486, 0.259)
Cortical porosity (%)	0.162 (−0.150, 0.475)	−0.192 (−0.502, 0.118)	−0.164 (−0.488, 0.160)	−0.257 (−0.616, 0.101)	−0.109 (−0.441, 0.223)	−0.208 (−0.585, 0.169)
Cortical VMS (MPa)	−0.237 (−0.537, 0.063)	**0.344 (0.055, 0.633)**	−0.006 (−0.327, 0.315)	−0.188 (−0.537, 0.161)	−0.254 (−0.566, 0.058)	**0.411 (0.061, 0.760)**
Estimated bone failure load (N)	0.125 (−0.174, 424)	−0.074 (−0.374, 0.226)	0.034 (−0.279, 0.347)	−0.147 (−0.490, 0.195)	0.212 (−0.095, 0.519)	0.025 (−0.341, 0.390)
Bone stiffness (N/mm)	−0.138 (−0.438, 0.163)	0.067 (−0.235, 0.369)	−0.063 (−0.378, 0.251)	0.123 (−0.223, 0.468)	−0.203 (−0.513, 0.106)	−0.061 (−0.428, 0.306)

All data adjusted for age, MVPA, BMI and vitamin D. Bold values are significant at *p* < 0.05. Abbreviations: DXA, dual-energy x-ray absorptiometry; HRpQCT, high-resolution peripheral quantitative computed tomography; EQ5D, EuroQol-5D; MFES, modified falls efficacy scale; SPPB, short physical performance battery; BMC, bone mineral content; aBMD, areal bone mineral density; LS, lumbar spine; TBS, trabecular bone score; FN, femoral neck; VMS, von Mises stress.
